# Use of Track One Prioritized Examination for Pharmaceutical Patents

**DOI:** 10.1001/jamahealthforum.2024.1886

**Published:** 2024-07-19

**Authors:** S. Sean Tu, William B. Feldman

**Affiliations:** 1 West Virginia University College of Law, Morgantown; 2 Division of Pharmacoepidemiology and Pharmacoeconomics, Department of Medicine, Brigham and Women’s Hospital and Harvard Medical School, Boston, Massachusetts; 3 Division of Pulmonary and Critical Care Medicine, Department of Medicine, Brigham and Women’s Hospital and Harvard Medical School, Boston, Massachusetts

## Abstract

This study examines the use of the Track One prioritized patent examination program by pharmaceutical manufacturers from 2011 to 2022.

## Introduction

Pharmaceutical manufacturers obtain patents at various points leading up to and following US Food and Drug Administration (FDA) approval of new drugs. Most pharmaceutical patents cover secondary features of products rather than active ingredients, with a rapidly growing share of continuations disclosing no new inventions beyond those covered in closely related original patents.^1,2^ Manufacturers seeking to maximize the effect of their patent portfolios may time additions in a way that creates uncertainty for generic and biosimilar firms, for example, by obtaining patents just as original statutory exclusivities granted by the FDA are set to expire. This can drive delays for generic or biosimilar competition because competitors become aware of a patent’s scope (and therefore what must be contested) only after the patent is granted.

A key tool that brand-name companies use to control patent timing is the Track One prioritized patent examination program.^3^ Patent applicants pay the US Patent and Trademark Office (USPTO) a fee for priority review within 1 year of obtaining Track One status, expediting a process that can often take several years.^4^ We analyzed all Track One patents granted from the pathway’s inception in September 2011 through December 2022 to understand how drug manufacturers have used prioritized review to build their patent portfolios.

## Methods

For this study, we filed a Freedom of Information Act request for all Track One patents granted from September 2011 to December 2022. We categorized patents in the FDA’s *Approved Products With Therapeutic Equivalence Evaluations* (known as the *Orange Book*) as Track One or non–Track One and generated a dataset of all litigated biologic patents using a legal database (Lex Machina). Although manufacturers of small-molecule drugs must list all key patents in the Orange Book, biologic manufacturers have no such listing requirement; therefore, we focused on litigated biologic patents as only these patents are publicly disclosed. This study was not submitted for institutional review board approval because it is based on publicly available data and involved no health records (45 CFR §46.101).

We determined when patents were granted relative to approval of the corresponding drug and whether the patent was a continuation or original patent (eMethods in Supplement 1). Biologics all receive 12 years of statutory exclusivity, but small-molecule drugs were analyzed separately based on the exclusivity type granted at approval: rare disease drugs (7 years), new chemical entities (5 years), and all other drugs (which can receive up to 3 years of exclusivity). We performed descriptive analysis to the examine timing of Track One vs non–Track One patents relative to the drug’s approval in Excel, version 16.8 (Microsoft).

## Results

**Figure 1.**
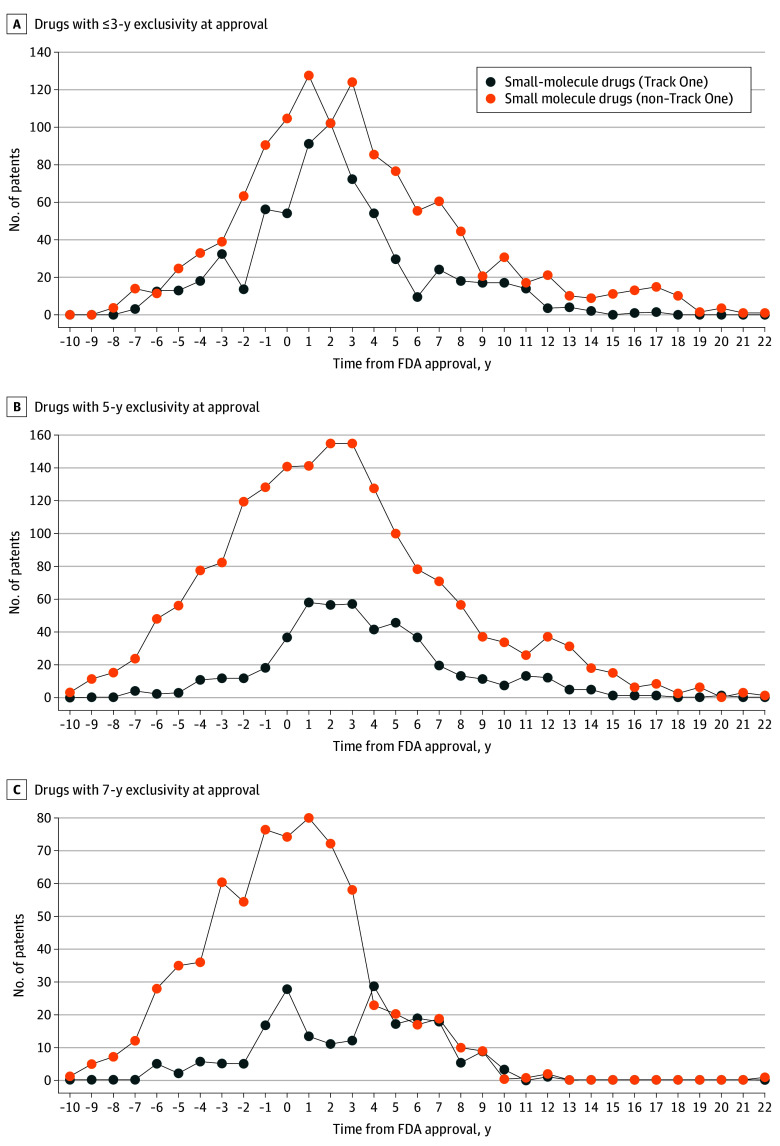
Track One vs Non–Track One Patents on Small-Molecule Drugs This figure shows the number of Track One (blue) and non–Track One (orange) patents granted on small-molecule drugs relative to approval by the US Food and Drug Administration (FDA). Patents are separately presented on small-molecule drugs with up to 3 years of statutory exclusivity in panel A (previously approved active ingredients with new studies completed), 5 years of statutory exclusivity in panel B (new chemical entities), and 7 years of statutory exclusivity in panel C (rare disease indications).

**Figure 2.**
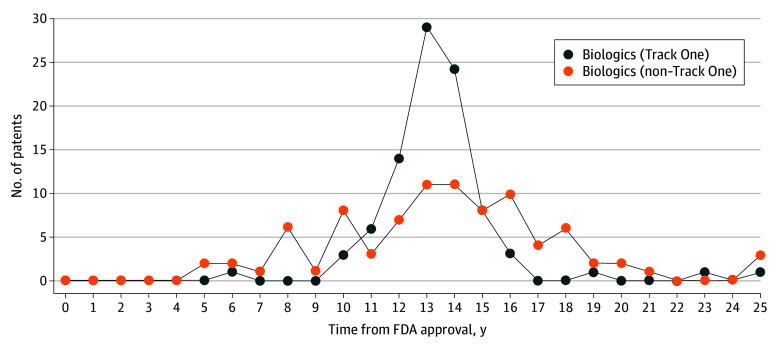
Track One vs Non–Track One Patents on Biologic Drugs This figure shows the number of Track One (blue) and non–Track One (orange) patents granted on biologic drugs relative to approval by the US Food and Drug Administration (FDA).

Track One patents comprised 27% (1350/5085) of all patents on small-molecule drugs and 51% (91/179) of patents on biologics; 86% (1155/1350) of Track One patents on small-molecule drugs and 90% (82/91) on biologics were continuation patents, compared to only 71% (2657/3735) of non–Track One patents on small-molecule drugs and 66% (58/88) on biologics. Track One patents peaked later for small-molecule drugs with 5- and 7-year exclusivities at approval (with peaks in years 3 and 4, respectively) compared with small-molecule drugs with shorter exclusivities (year 2) (Figure 1), peaking latest for biologic drugs (year 13) (Figure 2).

## Discussion

This study demonstrated that brand-name firms frequently obtain drug patents as statutory exclusivity periods expire and disproportionately rely on Track One review just ahead of these expirations to build their patent portfolios. Biologic manufacturers appear especially reliant on prioritized review. In part, this may be because shifting patent claims could cause longer delays for biosimilar firms, as they may be forced to conduct new studies going beyond those typically required for generic small-molecule drugs.

By delaying competition, unpredictability generated by Track One patents for both generic and biosimilar firms can keep prices high, potentially driving reduced adherence and worse health outcomes.^5^ One study limitation was not including less common USPTO priority review programs. To facilitate timely generic and biosimilar entry, the USPTO could consider allowing Track One status for only original patents, not continuations.^6^

## Notes

Supplement 1. eMethods. Supplementary Methods

Supplement 2. Data Sharing Statement
