# Supplementary material for: Use of Track One Prioritized Examination for Pharmaceutical Patents
Source: JAMA Health Forum. 2024 Jul 19;5(7):e241886. doi: 10.1001/jamahealthforum.2024.1886 (PMC11259899; doi:10.1001/jamahealthforum.2024.1886)
Supplement: Supplement 1. — eMethods. Supplementary Methods [file jamahealthforum-e241886-s001.pdf]

# Supplemental Online Content

Tu SS, Feldman WB. Use of Track One prioritized examination for pharmaceutical patents. *JAMA Health Forum*. 2024;5(7):e241886.
doi:10.1001/jamahealthforum.2024.1886

eMethods. Supplementary Methods

This supplemental material has been provided by the authors to give readers additional information about their work.

© 2024 Tu SS et al. *JAMA Health Forum.*

**eMethods**

Patent classification

Patents were classified as “continuation” or “original” using PatentAdvisor.com, a commercial database with patent prosecution information for all US patent prosecution along with filing and issue dates from 1976-present. Continuation patents include continuation, continuation-in-part, and divisional applications. This database has been used in prior studies of continuation patents.[1]

Exclusivity periods among small-molecule drugs

Small-molecule drugs were placed into one of three mutually exclusive categories based on statutory exclusivity periods at the time of approval: (1) small-molecule drugs with 7-year orphan drug exclusivities, (2) small-molecule drugs with 5-year new molecular entity exclusivities, and (3) small-molecule drugs eligible only for 3-year clinical investigation exclusivities. We first identified all drugs with orphan indications at the time of approval—using the FDA Orphan Drug Database[2]—which receive 7 years of statutory exclusivity. Only orphan indications at the time of approval were considered (orphan indications added later were excluded). We then identified all remaining drugs receiving type-1 approval from the Food and Drug Administration (FDA). Type 1 approvals are for new molecular entities, and are eligible for 5 years of market exclusivity.[3] All other products were classified as drugs eligible for only 3 years of statutory exclusivity (new clinical investigation exclusivity).

Timing of patent applications and issue dates relative to FDA approval

© 2024 Tu SS et al. *JAMA Health Forum.*

We determined filing dates and issue dates using PatentAdvisor.com. When a patent was listed on more than one drug, we used the first drug on which it was listed for analysis.

## References


1. Tu SS, Kesselheim AS, Wetherbee, Feldman W. Changes in the Number of Continuation Patents on Drugs Approved by the FDA Between 2000-2015. JAMA 330(5): 469-470 (2023).

2. Orphan Drug Designations and Approvals https://www.accessdata.fda.gov/scripts/opdlisting/oopd/index.cfm (Accessed February 14, 2024).

3. Frequently Asked Questions on Patents and Exclusivity https://www.fda.gov/drugs/development-approval-process-drugs/frequently-asked-questions-patents-and-exclusivity (Accessed February 14, 2024).


© 2024 Tu SS et al. *JAMA Health Forum.*
