# Supplementary material for: Use of Track One Prioritized Examination for Pharmaceutical Patents
Source: JAMA Health Forum. 2024 Jul 19;5(7):e241886. doi: 10.1001/jamahealthforum.2024.1886 (PMC11259899; doi:10.1001/jamahealthforum.2024.1886)
Supplement: Supplement 2. — Data Sharing Statement [file jamahealthforum-e241886-s002.pdf]

Data Sharing Statement

Tu. Use of Track One Prioritized Examination for Pharmaceutical Patents. *JAMA Health Forum*. Published July 19, 2024. doi:10.1001/jamahealthforum.2024.1886

Data
Data available: Yes
Data types: Data (not involving human participants)
How to access data: Shine.tu@mail.wvu.edu
When available: With publication

Supporting Documents
Document types: Statistical/analytic code
How to access documents: shine.tu@mail.wvu.edu
When available: With publication

Additional Information
Who can access the data: Anyone who wishes to have the data can obtain the data by e-mailing shine.tu@mail.wvu.edu
Types of analyses: For any purpose
Mechanisms of data availability: Anyone who wishes to have the data can obtain the data by e-mailing shine.tu@mail.wvu.edu
